# Barriers and Facilitators to Resuming In-Person Psychotherapy with Perinatal Patients amid the COVID-19 Pandemic: A Multistakeholder Perspective

**DOI:** 10.3390/ijerph182212234

**Published:** 2021-11-22

**Authors:** Nicole Andrejek, Sabrina Hossain, Nour Schoueri-Mychasiw, Gul Saeed, Maral Zibaman, Angie K. Puerto Niño, Samantha Meltzer-Brody, Richard K. Silver, Simone N. Vigod, Daisy R. Singla

**Affiliations:** 1 Lunenfeld Tanenbaum Research Institute, Sinai Health, Toronto, ON M5G 1X5, Canada; nicole.andrejek@sinaihealth.ca (N.A.); Sabrina.Hossain@sinaihealth.ca (S.H.); Nour.Schoueri-Mychasiw@sinaihealth.ca (N.S.-M.); Maral.Zibaman@sinaihealth.ca (M.Z.); Angie.PuertoNino@sinaihealth.ca (A.K.P.N.); 2 Dalla Lana School of Public Health, University of Toronto, Toronto, ON M5T 3M7, Canada; gul.saeed@mail.utoronto.ca; 3 Department of Psychiatry, School of Medicine, University of North Carolina, Chapel Hill, NC 27514, USA; samantha_meltzer-brody@med.unc.edu; 4 Department of Obstetrics & Gynecology, NorthShore University Health System, Evanston, IL 60201, USA; RSilver@northshore.org; 5 Department of Psychiatry, Women’s College Hospital, Toronto, ON M5S 1B2, Canada; simone.vigod@wchospital.ca; 6 Department of Psychiatry, Temerty Faculty of Medicine, University of Toronto, Toronto, ON M5T 1R8, Canada; 7 Campbell Family Mental Health Research Institute, Center of Addiction and Mental Health, Toronto, ON M6J 1H4, Canada

**Keywords:** perinatal depression and anxiety, psychotherapy, barriers, facilitators, COVID-19

## Abstract

During the COVID-19 pandemic, outpatient psychotherapy transitioned to telemedicine. This study aimed to examine barriers and facilitators to resuming in-person psychotherapy with perinatal patients as the pandemic abates. We conducted focus group and individual interviews with a sample of perinatal participants (*n* = 23), psychotherapy providers (*n* = 28), and stakeholders (*n* = 18) from Canada and the U.S. involved in the SUMMIT trial, which is aimed at improving access to mental healthcare for perinatal patients with depression and anxiety. Content analysis was used to examine perceived barriers and facilitators. Reported barriers included concerns about virus exposure in a hospital setting (77.8% stakeholders, 73.9% perinatal participants, 71.4% providers) or on public transportation (50.0% stakeholders, 26.1% perinatal participants, 25.0% providers), wearing a mask during sessions (50.0% stakeholders, 25.0% providers, 13.0% participants), lack of childcare (66.7% stakeholders, 46.4% providers, 43.5% perinatal participants), general transportation barriers (50.0% stakeholders, 47.8% perinatal participants, 25.0% providers), and the burden of planning and making time for in-person sessions (35.7% providers, 34.8% perinatal participants, 27.8% stakeholders). Reported facilitators included implementing and communicating safety protocols (72.2% stakeholders, 47.8% perinatal participants, 39.3% providers), conducting sessions at alternative or larger locations (44.4% stakeholders, 32.1% providers, 17.4% perinatal participants), providing incentives (34.8% perinatal participants, 21.4% providers, 11.1% stakeholders), and childcare and flexible scheduling options (31.1% perinatal participants, 16.7% stakeholders). This study identified a number of potential barriers and illustrated that COVID-19 has fostered and amplified barriers. Future interventions to facilitate resuming in-person sessions should focus on patient-centered strategies based on empathy regarding ongoing risk-aversion among perinatal patients despite existing safety protocols, and holistic thinking to make access to in-person psychotherapy easier and more accessible for perinatal patients.

## 1. Introduction

Recent studies suggest that approximately 8 to 13% of women experience perinatal depression in Canada and the United States [1,2], and 15–20% of women experience perinatal anxiety [3]. Psychotherapy has been shown to be effective in treating the significant burden of perinatal depression and anxiety [4]; however, as few as 12% of women with perinatal depression access mental healthcare services [5]. The COVID-19 pandemic has further exacerbated depression and anxiety symptoms among perinatal populations [6,7].

During the pandemic, many medical interventions—including in-person psychotherapy with perinatal patients—were postponed, stopped, or altered to avoid in-person contact among staff, patients, and participants [8,9]. To continue providing care, in-person psychotherapy sessions quickly transitioned to telemedicine [10]. Although there is a growing body of evidence that suggests that telemedicine is a convenient [11], efficacious [12,13], cost-effective [14], and an acceptable [12,15] mode of receiving psychotherapy, there remains a need for further evidence to decipher whether psychotherapy delivered via telemedicine is as effective as receiving it in-person [16,17], and for whom telemedicine may be most suitable [18].

Although telemedicine has been an important avenue to deliver psychotherapy during the pandemic, reliance on telemedicine may create new and unforeseen barriers [19]. For instance, we anticipate that some perinatal patients may encounter specific challenges when participating in psychotherapy via telemedicine that may impact their experiences and the quality of the care they receive, such as a lack of privacy and competing demands at home. These potential barriers may be amplified for those who have multiple children being homeschooled and spouses working from home due to the pandemic. Furthermore, some research suggests that many individuals simply prefer in-person treatment, and may choose not to receive care if in-person sessions are not an option for them [20]. Therefore, given the need for further clinical research on the effectiveness of telemedicine among perinatal patients [16,17,18], the general need to increase and improve avenues to accessing psychotherapy for perinatal patients [4], and with the recent rollout of COVID-19 vaccines, it is critical, ethical, and timely to examine barriers and facilitators to resuming in-person psychotherapy among perinatal patients, providers, and other relevant stakeholders.

In consideration of resuming in-person sessions within the context of COVID-19, clinicians and clinical researchers may find that perinatal patients are a reluctant population because they may feel that they are a particularly vulnerable population [21]. Consequently, perinatal patients may be more fearful than other populations about contracting COVID-19 while pregnant or exposing their newborn [21]. Therefore, in order to resume in-person psychotherapy research and practice with perinatal patients, it is necessary to consider potential barriers and facilitators in the current context through a qualitative, multistakeholder approach that includes the perspectives of perinatal patients, psychotherapy providers who work with this population, and relevant community- and hospital-based stakeholders to inform patient-centered policies and best practices. This approach will provide perspectives from a range of individuals with first-hand experience and expertise who can identify barriers and facilitators that might be prevalent among perinatal patients within the context of COVID-19 and those that would exist regardless of the pandemic.

Therefore, in this qualitative study, our primary aim was to examine the barriers that might impact perinatal patients’ access to in-person psychotherapy sessions and facilitators to resuming in-person sessions from the perspectives of perinatal patients, providers, and relevant community- and hospital-based stakeholders. The current study was conducted within the *Scaling Up Maternal Mental healthcare by Increasing access to Treatment* (SUMMIT) trial, which is aimed at increasing access to mental health treatment among perinatal patients with symptoms of depression and anxiety in Canada and the United States by delivering a form of psychotherapy called behavioral activation (BA) [22]. Though a central aim of the SUMMIT trial is to compare whether BA delivered via telemedicine is as effective as when it is delivered in-person, similar to many other clinical research and practices, all perinatal participants in the SUMMIT trial were exclusively randomized to telemedicine beginning in mid-March 2020 due to COVID-19. This study included two objectives: (1) to identify barriers that might impact perinatal patients’ access or decision to attend in-person psychotherapy and (2) to explore facilitators to resume in-person psychotherapy sessions with perinatal patients.

## 2. Materials and Methods

### 2.1. Study Sites and Ethics

SUMMIT is a multi-site, four-arm, randomized, non-inferiority trial that is being conducted across three hubs in Toronto, Canada and in Chapel Hill and Chicago in the United States. In Toronto, the larger SUMMIT trial recruited perinatal patients from the Sinai Health System, Women’s College Hospital, and St. Michael’s Hospital—three major academic hospitals that are all affiliated with the University of Toronto and operate via various referral pathways, such as patients’ family doctors, obstetricians, or psychiatrists. In North Carolina, perinatal participants were recruited from three clinical sites affiliated with the University of North Carolina Women’s and Neuroscience Hospitals. In Chicago, perinatal participants were recruited from fourteen affiliated obstetric and family medicine clinics. For more information, full details of the SUMMIT trial protocol have been published elsewhere [22].

Ethical approval was obtained from Clinical Trials Ontario Research Ethics Board (1895), Institutional Review Board of NorthShore University HealthSystems [EH18-129], and UNC Biomedical Institution Review Board (19-1786).

### 2.2. Sample

The sample for this study included perinatal, provider, and stakeholder participants. First, perinatal participants were identified and then recruited for interviews in this current study from the SUMMIT trial, which recruited individuals who were 18 years of age or older and were either pregnant (up to 36 weeks) or postpartum (4 to 30 weeks) with depressive symptoms (EPDS ≥ 10). Exclusion criteria for the larger trial included non-English or non-Spanish speakers, individuals with active suicidal intent, active symptoms of psychosis or mania, and/or active substance abuse or dependence, individuals whose psychotropic medication dose or medication changed within two weeks of enrolment or beginning the BA sessions, individuals who are in ongoing psychotherapy, and individuals who experienced severe fetal anomalies, stillbirth, or infant death at the time of enrolment for index pregnancy [22]. In the SUMMIT trial, perinatal patients participate in 6–8 behavioral activation (BA) psychotherapy sessions. BA focuses on reducing symptoms of depression and anxiety by engaging in enjoyable or fulfilling activities that align with patients’ values, and has been shown effective in reducing symptoms of depression [22].

To be eligible to participate in our study, perinatal participants must have consented to be contacted to participate in qualitative research in the SUMMIT trial and must have completed at least 5 of the 6–8 BA sessions. Due to COVID-19, all perinatal participants in the current study received BA sessions via telemedicine. Among consenting perinatal participants, the SUMMIT research team identified and contacted a randomly selected subset of eligible perinatal participants willing to participate in this current qualitative study.

Second, the provider group consisted of non-specialist providers (NSPs) and specialist providers (SPs) who delivered the BA sessions in the SUMMIT trial. NSPs were nurses and midwives with no previous mental health expertise that received training to deliver BA. SPs were psychotherapists, psychologists, psychiatrists, and social workers. To be eligible to participate in the current study, providers must have consented to participate in qualitative research through the SUMMIT research team who used convenience sampling to recruit and invite providers to participate in this current study via email.

Third, the stakeholder group comprised of *community-based stakeholders*, including patient advocates, clinicians, community partners, and study consultants, and *hospital-based stakeholders*, which included psychiatrists, hospital administrators, and psychotherapists that SUMMIT researchers recruited from the trial’s three research sites. These stakeholders were included due to their robust knowledge, expertise, and experience working with patients both before and during the pandemic in relevant settings, such as in perinatal clinics and the hospitals where SUMMIT conducts the BA sessions, and because they have firsthand experience with the barriers that patients encounter and ways to facilitate resuming in-person activities. Stakeholders were contacts of the diverse SUMMIT team across study sites and were recruited by convenience sampling. Once recruited, the SUMMIT research team invited them to participate in this current study via email.

### 2.3. Data Collection

Data collection took place between August 2020 and March 2021. We conducted semi-structured individual and focus group interviews to examine our participants’ perspectives on what they foresee might be future barriers to resuming in-person psychotherapy sessions with perinatal patients. Then, we asked all participants their perspectives about potential facilitators to resuming in-person psychotherapy sessions with perinatal patients (see Supplementary File S1 for a sample of the interview questions). We prompted our participants to discuss potential COVID-19-specific barriers and facilitators and general barriers and facilitators (those that might exist regardless of the pandemic) to resuming in-person sessions with perinatal patients. Two trained qualitative researchers (N.A. and S.H.) conducted all individual and focus group interviews using the HIPPA/PHIPA-compliant version of Zoom^TM^.

For provider data collection, the research team conducted eight focus group interviews with provider participants, with a range of two to six providers in each group. The research team also conducted individual interviews with three providers who were unable to attend the focus groups. For stakeholder data collection, we conducted one focus group with nine stakeholders and separately conducted nine individual interviews with stakeholders. For perinatal participant data collection, we conducted individual interviews with perinatal participants in order to maintain confidentiality and explore their perspectives on barriers and facilitators to attending in-person psychotherapy during the pandemic. Interviews were conducted until saturation was reached, which refers to the point in the data collection process in which the qualitative researchers assess that they have reached consensus and that no new themes or codes were emerging in the data [23].

Participants either signed a paper consent form or an online consent form via REDCap™. The researchers obtained both written and verbal consent before conducting all individual and focus group interviews and gave full details of the study prior to beginning interviews. Interviews and focus groups lasted approximately 40 to 60 min. All participants received an e-gift card ($15 USD or $20 CAD) as remuneration for their participation in the interviews. All participants were assigned an identification code, and any identifying details were obscured to maintain confidentiality.

### 2.4. Data Analysis

Trained research assistants audio-recorded the interviews, encrypted them for security, transcribed them verbatim, and checked their quality. Qualitative data were analyzed using NVivo™. Using previously established methodologies [24], we conducted a content analysis to identify common barriers and facilitators to resuming in-person sessions. We calculated the inter-rater reliability using the Kappa (κ) coefficient (κ = 0.95), indicating a substantial agreement between the authors. We used an iterative coding process that identified patterns across participants’ responses through two stages of coding: an initial coding stage and a secondary, focused coding stage. For the purpose of this study, our analysis focused on reported barriers and facilitators to accessing in-person psychotherapy amid the pandemic. In addition, we calculated descriptive results (means, ranges, and frequencies) for relevant demographic characteristics. We derived perinatal and provider participant descriptive data from a demographic questionnaire that they completed prior to participating in interviews (see Supplementary File S2 for demographic questionnaire). Stakeholder demographic data were not collected.

## 3. Results

Our results describe the qualitative data collected from perinatal participants (*n* = 23), providers (*n* = 28), and a range of stakeholders (*n* = 18), across Canada and the United States (see Table 1 for participant characteristics).

### 3.1. Perceived Barriers to Resuming In-Person Psychotherapy

All participant groups were asked about barriers to resuming in-person psychotherapy with perinatal patients. Participants responded with a number of perceived COVID-19-specific and general barriers (those that would exist during and after the pandemic). The barriers to resuming in-person psychotherapy sessions in the future that were specific to the COVID-19 pandemic were (1) concerns about virus exposure in a hospital setting, (2) concerns about virus exposure while taking public transportation, and (3) concerns about having to wear a mask during psychotherapy sessions (see Table 2). The general barriers reported that are not specific to—but may be exacerbated by—the pandemic were (1) lack of childcare, (2) general transportation barriers, and (3) planning and scheduling to attend in-person sessions at a hospital while navigating other priorities as a busy new mother.

#### 3.1.1. COVID-19 Barriers: Concerns about Safety and Virus Exposure while in the Hospital

Of the barriers reported by our participants that are specific to the COVID-19 pandemic, the majority of perinatal participants (*n* = 17 of 23, 73.9%), providers (*n* = 20 of 28, 71.4%), and stakeholders (*n* = 14 of 18, 77.8%) expressed that future participants may be reluctant to attend in-person BA sessions due to concerns about virus exposure in a hospital setting. These participants described that pregnant and postpartum patients might be particularly fearful of potentially contracting COVID-19 and transmitting it to their newborn and their families, especially at a hospital. For instance, a participant expressed that:

*“If you have a new baby or are pregnant, you won’t want to expose yourself [at the hospital to COVID-19] or expose your new baby. [In the trial patients will] all be pregnant or have babies. So, it won’t just be us being at risk, we’ll probably have to bring our infant with us too”*.

(Perinatal_02_ US)

A provider also explained that:

*“Now that we’re in COVID, there’s going to be a lot of anxiety… Even [if they do not bring their] kids, my general sense is that there’s a fear of a hospital setting. I think that’s going to be a barrier... These waiting rooms are huge, packed, full of people, [which may be a deterrent for future participants]”*.

(NSP_06_Canada)

Some perinatal participants expressed that if they were randomized to receive in-person sessions; they would be concerned about their safety at the hospital and choose to opt out:

*“I would be worried about COVID transmission, especially postpartum… I don’t think [future perinatal participants] would be very comfortable… I would not be very comfortable bringing my child into a session… So, if I had to go to the hospital in-person [for BA sessions], I would have chosen not to”*.

(Perinatal_12_ Canada)

#### 3.1.2. COVID-19 Barriers: Concerns about Virus Exposure while Taking Public Transportation

The second COVID-19-specific barrier was that perinatal participants (*n* = 6 of 23, 26.1%), providers (*n* = 7 of 28, 25.0%), and stakeholders (*n* = 9 of 18, 50.0%) described that future patients may be reluctant to attend in-person sessions because of the potential COVID-19 exposure while taking public transportation. One perinatal woman explained that:

*“I’m very risk-averse…I’m not sure at what point I would feel comfortable taking the subway to come to [Canadian hospital], which is what I did before [the pandemic]... I don’t know when I would feel comfortable doing that, to be honest”*.

(Perinatal_15_ Canada)

Likewise, a U.S. provider stated that: *“Transportation [would be a barrier] for clients who don’t have a car… [and] having to risk exposure on the bus or the train to get to the appointment”* (SP_07_US). One Canadian stakeholder highlighted that this would be a barrier especially in the winter: *“We have to think about our winters [since more people take transit in the winter rather than walking] and the risk of being on a very crowded transit”* (Stakeholder_03_Canada).

#### 3.1.3. COVID-19 Barriers: Wearing a Mask during Psychotherapy Sessions

The third COVID-19-specific barrier endorsed by our participants was needing to wear masks during BA sessions (perinatal participants *n* = 3 of 23, 13.0%; providers *n* = 7 of 28, 25.0%; and stakeholders *n* = 9 of 18, 50.0%). Overall, participants expressed concern that wearing masks might impact the quality and experience of the BA sessions: *“[An] obstacle [is] that it’s hard to imagine doing therapy in masks. It’s probably going to make it so impersonal. I would not want to do that*” (Perinatal_04_ Canada); “*If [the patient] is required to wear a mask in the common areas or even throughout the entire therapy, how does that feel to her? Is she okay with wearing a mask? Does that induce her anxiety?*” (Stakeholder_05_Canada);

*“I can’t really see us doing in-person care until we don’t have to wear masks. I don’t know if [perinatal patients] will feel comfortable…especially if they have to bring their baby with them. [Providers] are used wearing [masks] all the time [but perinatal patients aren’t]”*.

(NSP_04_US)

#### 3.1.4. General Barriers: Lack of Childcare

Of the barriers reported by our participants that are not uniquely specific to the COVID-19 pandemic, perinatal participants (*n* = 10 of 23, 43.5%), providers (*n* = 10 of 28, 46.4%), and stakeholders (*n* = 12 of 18, 66.7%) expressed that future perinatal patients may be reluctant to attend in-person BA sessions because they do not have childcare: *“This barrier is not COVID-related; it’s just always been a barrier that you can’t find childcare”* (Stakeholder_12_Canada).

Although our participants expressed that childcare would be a challenge regardless of the pandemic, they asserted that regulations and safety concerns due COVID-19 has exacerbated this problem. For instance, there have been many periods during the pandemic in which children were not in school, daycares were closed, and babysitters or family members were unable to provide ad-hoc childcare. One perinatal participant said that “*maybe post-COVID it would be different, but we have no help [with the kids]. So, there’s no way I would have the time and ability to [go in-person] on a regular basis.”* (Perinatal_06_US). Similarly, a provider stated that, “*we had childcare at [the hospital]”* that parents could leave their kids at during psychotherapy sessions but that this was closed due to COVID-19 and explained that *“I have no idea when that would be able to be opened up. I feel like it would be very long [in the future]. So, finding other childcare would be a big barrier*” (SP_06_Canada).

#### 3.1.5. General Barriers: Transportation (Traffic, Drive Time, no Access to Vehicle)

Perinatal participants (*n* = 11 of 23, 47.8%), providers (*n* = 7 of 28, 25.0%), and stakeholders (*n* = 9 of 18, 50.0%) also expressed that issues related to transportation, such as long drive times, traffic, not having access to a vehicle, and cost of parking, would be barriers for future perinatal patients to attend in-person sessions, regardless of the pandemic. One perinatal participant explained that “*COVID aside, I’m [not in the same city as the hospital], so it’s quite a commute. And then you’re dealing with traffic and paying for parking. Those would be barriers for me”* (Perinatal_02_ Canada). A U.S. provider also said that:

*We have a lot of patients who have transportation [barriers] [and may not] even agree to participate in therapy because they can’t imagine how they would even get there if they’re a one-car family or a no-car family or they don’t want to take the bus with a child*.

(SP_05_US)

One stakeholder highlighted that this barrier may be even more salient for low-income perinatal patients:

*We don’t have great public transportation options [in our city] because we’re much more spread out… and particularly for our lower income women, where you’re dealing with issues around cost and gas and all of those kinds of things, transportation will always be a barrier*.

(Stakeholder_20_US)

#### 3.1.6. General Barriers: Planning and Time Constraints for Busy New Moms

In addition to the barriers of finding childcare and the challenges of transportation, some of our perinatal participants (*n* = 8 of 23, 34.8%), providers (*n* = 13 of 28, 35.7%), and stakeholders (*n* = 5 of 18, 27.8%) stated that new mothers are particularly busy and have many time constraints in general, which may prevent them from accessing in-person therapy. Planning and finding the time in their schedules to attend in-person sessions might be a barrier that is particularly relevant for perinatal populations, who are often managing multiple priorities and have many demands on their time:

*A barrier is the busyness factor of new moms: trying to schedule it at a time where you don’t have your other children or during school hours, and then trying to avoid nap schedules. It’s tricky to get out the door and schedule things around your [other priorities], especially if you’re a working mom—that would be impossible*.

(Perinatal_23_Canada)

Similarly, a stakeholder explained that: “*I think one piece that doesn’t have to do with COVID at all, is just that pregnant and postpartum women are busy and have various competing demands, and it’s hard to get them to come in-person in general*” (Stakeholder_13_Canada). A provider echoed this sentiment and stated that, “*new moms have so many responsibilities… so they would have to multitask a lot [to come in-person] or just not have therapy*” (NSP_02_US).

### 3.2. Perceived Facilitators to Resuming In-Person Psychotherapy

All participant groups were asked about facilitators to resuming in-person psychotherapy with perinatal patients. The facilitators to resuming in-person psychotherapy sessions in the future that were specific to the COVID-19 pandemic were (1) implementing and communicating robust safety protocols and (2) having sessions at an offsite location, not so deep within the hospital, or in larger rooms (see Table 3). Our participants also suggested two facilitators that were not specific to the pandemic: (1) providing incentives, such as transportation or parking vouchers, and (2) providing childcare options and having flexible scheduling for session.

#### 3.2.1. COVID-19 Facilitators: Implementing and Communicating Robust Safety Protocols

Implementing and effectively communicating robust safety protocols was a facilitator suggested by 11 (47.8%) perinatal participants, 11 (39.3%) providers, and 13 (72.2%) stakeholders. Participants suggested that information could be disseminated to patients to address patients’ anxiety about coming to the hospital. A provider said, “*we’d have to be able to provide some kind of assurance of safety in educational materials that were provided to the patients along with their* *SUMMIT orientation, explaining why in-person was a safe option*” (NSP_01_US). Perinatal and stakeholder participants also suggested the following:

*“Definitely let the patient know [what] the plans [is for] cleanliness, ‘this is what we do, this is our procedure, you don’t have to see anyone, you just come in, you don’t have to touch anything, we’re going to be 6 feet apart, there is a partition [or] Plexiglas.’ Just reassuring them on that end”*.

(Perinatal_01_US)

Specifically, a stakeholder suggested that:

*“A way to decrease the fear and anxiety is to create a little info booklet or package that we can give to people that are consenting to in-person... [on] the use of masks and all of the stuff…to help reduce any of those issues”*.

(Stakeholder_07_Canada)

#### 3.2.2. COVID-19 Facilitators: Conducting Sessions at Offsite Locations, Not so Deep within the Hospital, or in Larger Rooms

Another facilitator suggested by 4 (17.4%) perinatal participants, 9 (32.1%) providers, and 8 (44.4%) stakeholders was to provider alternative locations to where sessions are delivered, such as rooms that are not so deep inside the hospitals or in larger rooms where participants can socially distance or be separated by Plexiglas. For instance, participants suggested that holding sessions in a larger room could address barriers to attending in-person psychotherapy, such as concerns about having to wear masks: “*Maybe [have sessions] in a larger room where you can distance even more than six feet apart*” (Stakeholder_17_Canada).

Participants suggested that conducting the BA sessions on the first floor of the hospital, or even offsite at a non-clinical location, may be a facilitator for resuming in-person psychotherapy sessions: “*I think patients would be more interested in coming to some sort of outpatient facility or not the hospital, like a private office somewhere … Of course, there’s cost implications and cleaning and all these things [to consider]*” (SP_07_US).

A perinatal participant also suggested that:

*“Instead of walking through a busy lobby, taking the elevator up, [I’d prefer] a room on the first floor as close to the door as possible [and] one person [goes in] at a time. That would be great… Walk[ing] through a healthcare facility… would be super nerve-wracking…I had to take an elevator [to see my doctor], and I was panicked for three days after”*.

(Perinatal_23_ Canada)

#### 3.2.3. General Facilitators: Providing Incentives

In order to mediate general transportation barriers, such as long drive times, traffic, not having access to a vehicle, and cost of parking, 8 (34.8%) perinatal participants, 6 (21.4%) providers, and 2 (11.1%) stakeholders suggested providing incentives, such as transportation or parking vouchers. A perinatal patient explained that: “*gas and parking is so expensive, so maybe if there’s free parking or a parking voucher [that would an incentive].*” Similarly, one of the stakeholders expressed that *“For some, it’s a financial burden [to travel]. So, paying for parking if they’re coming from far away [would reduce that barrier]* (Stakeholder_14_Canada). A U.S. provider also emphasized that a “*taxi, bus token, or parking vouche*r” would be a particularly beneficial incentive and would help reduce the transportation-cost barrier “*for lower-income folks*” who may find such costs particularly burdensome.

#### 3.2.4. General Facilitators: Childcare and Flexible Scheduling for Sessions

To further reduce barriers for perinatal patients to attend in-person therapy, 9 (31.1%) perinatal participants and 3 (16.7%) stakeholders suggested that there will be a need to increase accessibility to therapy by providing childcare at the hospital and having flexible time slots for sessions. Although this theme was reported by fewer participants, participants who did report this theme provided practical and creative solutions to improving access for perinatal patients to resume in-person sessions, such as allowing perinatal patients to bring children with them, resuming on-site daycare, and having flexible or extended session times to allow for perinatal patients to work around their families’ schedules. For instance, one U.S. perinatal participant said that: “*If you had somewhere where you could bring your children or if the mom could bring her infant and children with her and somebody there or right next door [is there] to help watch, that would be very beneficial*” (Perinatal_06_US).

One stakeholder also suggested working with the patient to schedules times when they are already coming into the hospital to make attending sessions feel less burdensome in their busy schedule:

*“Another [thing that can be done is ask]: does the patient already have visits that they need to come to for other reasons that we couple them together? So that [the patient] can be like, ‘hey, I have to go into [the hospital/clinic] anyway to pick up a prescription (or whatever it is), and then I can just do this, so I’m not having to come twice?’”*.

(Stakeholder_17_Canada)

## 4. Discussion

We examined perceived future barriers to resuming in-person psychotherapy sessions with perinatal participants amid the COVID-19 pandemic. We anticipated that examining perceived barriers would produce important insights to perinatal patients’ decision-making practices regarding accessing in-person care. We then examined potential facilitators to resuming in-person psychotherapy sessions with perinatal patients with symptoms of depression and anxiety.

A key finding that emerged was that the majority of perinatal, provider, and stakeholder participants in the current study expressed a number of barriers to resuming in-person psychotherapy sessions that were specific to the COVID-19 pandemic, which included perinatal participants’ concerns about virus exposure in a hospital setting, virus exposure while taking public transportation, and having to wear a mask during psychotherapy sessions. Participants also expressed that there would be key barriers for perinatal patients regardless of the pandemic. These barriers were lack of childcare, general transportation barriers such as not having access to a vehicle and the high cost of parking at hospitals, and the burden of planning and making time to attend in-person sessions at a hospital while navigating other priorities. Based on participants’ responses about these barriers that might exist regardless of the pandemic, it became apparent that barriers, such as a lack of childcare, have been amplified by the ongoing pandemic.

### 4.1. Overcoming Challenges to Resuming In-Person Psychotherapy Sessions

Perinatal participants, providers, and stakeholders all suggested two ways to facilitate resuming in-person psychotherapy sessions that are related to the aforementioned barriers that were specific to COVID-19. The first suggested strategy was to implement and clearly communicate robust safety protocols to patients that is supported by research, health officials, and policy advisors across the globe, emphasizing the importance of basic safety protocols, such as requiring masks, promoting social distancing in spaces such as public transportation, and employing hand hygiene when accessing public spaces [25,26].

While many safety procedures are already in place at healthcare sites, our findings suggest that patients, and potentially their providers, may not be well informed about them or these procedures may feel unclear or difficult to access to patients. Thus, finding ways to clearly and effectively communicate safety protocols to perinatal patients and providers will be of particular importance. These findings are consistent with previous research that underscores that patients and providers tend to feel overwhelmed when unclear messages and guidelines about health and safety exist [27,28]. Studies have also shown that individuals with depression and anxiety are particularly risk-averse and reluctant to participate in clinical research or accessing healthcare that they perceived to be non-essential during a public health crisis, such as COVID-19 [29,30]. 

Our solution-focused findings confirm that clear, patient-centered communication strategies will be key to informing best practices and policies when resuming in-person psychotherapy sessions amid the COVID-19 pandemic. Patient-centered communication has been shown to help improve trust in healthcare institutions, services, and providers [31]. A patient-centered approach is also particularly important for healthcare services that focus on populations who may be more mistrusting or risk-averse, such as perinatal patients in our case [32]. We also anticipate that these findings will be applicable to clinicians working with other groups, including vulnerable populations, such as elderly and immunocompromised individuals, non-perinatal patients with depression and anxiety, and parents of young children who are unable to receive a COVID-19 vaccine and may be concerned about virus transmission to their children [21].

Based on our study and population, we propose that some of the best communication practices and strategies may include providing physical information sheets and access to a website or an online PDF outlining what patients can expect when they go to in-person psychotherapy sessions (Available online: https://www.flipsnack.com/E88FE7AA9F7/in-person-brochure_provider.html accessed on 22 May 2021). This information should include clearly outlined safety protocols written in straightforward terms, and should ideally be available in multiple languages when applicable. Other strategies may include providing patients with clear instructions about where to go within the hospital, allowing perinatal patients to wait outside or in their car where they can be called or sent a text message to avoid busy waiting rooms, to clearly articulate whether perinatal patients are allowed to bring infants and children to the location of their sessions, whether the location and room are stroller-friendly and large enough to fit patients and strollers in light of social distancing requirements, having COVID-19 screening calls prior to patients traveling to their sessions and guidelines on what to do if screens are positive, and incorporating non-punitive protocols for cancellations [9,25,26]. When resuming in-person psychotherapy sessions, clinical trials and clinicians in hospital, clinics, and private practice settings should be fully prepared with the necessary personal protective equipment, space, and protocols to reduce the likelihood of delays that might cause further anxiety and mistrust in individuals accessing in-person psychotherapy.

The second suggested strategy was to conduct psychotherapy not deep within the hospital setting, and also in larger-sized rooms. This suggestion may not be feasible for some psychotherapist clinicians and clinical studies. Nonetheless, perinatal patients may feel a sense of heightened anxiety of being exposed to COVID-19 while pregnant or exposing their newborn, and may also be concerned about having to wear a mask during sessions in general or needing to briefly remove their mask to use a tissue if they cry during sessions [21]. Therefore, if in-person sessions are in larger rooms, then perinatal patients may feel safer to socially distance. To avoid needing to wear masks, using Plexiglas barriers might be useful if possible. Moreover, if clinicians are able to hold sessions in offices outside of a hospital setting, this may help patients feel safer than navigating a large hospital, where fears of being in proximity to individuals with COVID-19 may arise.

In addition to the COVID-19-specific facilitators, our participants suggested two ways to facilitate resuming in-person psychotherapy sessions to overcome general barriers to transportation costs and the burden of seeking childcare and having to plan and make time to participate in in-person psychotherapy. First, our participants suggested providing incentives, such as transportation or parking vouchers, that may lessen some of the financial transportation burdens. This facilitator is particularly relevant for clinical researchers who may have access to funding. Clinicians aiming to improve access to perinatal mental healthcare may also want to work with hospital leaders and administrators to find solutions to minimizing the financial burdens for perinatal patients visiting the hospital. Although our results pertain to perinatal patients, this suggestion is useful and relevant for providers working with patients who are low-income to whom the burden of paying high costs for parking may make them less likely to access in-person care.

Perinatal participants and stakeholders also suggested improving access to in-person psychotherapy by providing childcare and holding extended or flexible time options for sessions. Informing perinatal patients that they are able to bring their infants with them to sessions would minimize the barrier of finding childcare, ensuring that there is a private, safe, and comfortable space for perinatal patients to breastfeed, and having flexible scheduling options to patients can work around when they have access to childcare are all straightforward, patient-centered recommendations to facilitate resuming in-person sessions with this population [33]. As the pandemic abates, offering onsite daycare would also further minimize some of the burdens of finding and planning for childcare.

Although the barriers and facilitators discussed in this study directly pertain to resuming in-person psychotherapy within the SUMMIT trial, they can also be extrapolated to other clinicians and clinical research trials that are aiming to resume in-person psychotherapy sessions. Similarly, our findings can be used to inform practices to make in-person psychotherapy more patient-centered for perinatal patients with symptoms of depression and anxiety. This approach aims to make health equity for perinatal patients with symptoms of depression and anxiety a priority in research and practice [34].

Furthermore, COVID-19 may continue to impact patients’ and study participants’ decisions to attend in-person psychotherapy, but so might the normalization and benefits of virtual care. Due to the pandemic, telemedicine became a common practice for delivering psychotherapy [8,10,13]. However, there is a need to further examine the important question of whether telemedicine-based psychotherapy sessions are as effective as in-person psychotherapy for perinatal patients with depression and anxiety. Therefore, creative problem solving to address potential barriers will be essential to resuming the SUMMIT trial, as well as other clinical research. Despite the benefits of telemedicine for many individuals, it is important to not assume that telemedicine is a viable or desirable option for all perinatal patients, especially those who may experience challenges of finding private space at home, may not have quality access to technology or Internet, or who may simply prefer to see their psychotherapy provider in-person [20]. Research suggests that few perinatal patients with depression access mental health services [5]; thus, a primary objective for clinicians and clinical researchers should be to endeavor to increase avenues and access to psychotherapy for perinatal patients with depression and anxiety.

### 4.2. Limitations and Strengths

This study has a few limitations. The first limitation is that we did not collect demographic data for our stakeholder group. Second, given our participants were living in different jurisdictions, our findings cannot account for how variations in local government regulations impacted our participants’ responses, such as the Stage two and Stage three reopening in Toronto in late summer and fall of 2020, and subsequent lockdown order at the end of November 2020. Third, future research should also examine how socio-demographic factors might impact perinatal patients’ attitudes and beliefs about barriers and facilitators to transitioning to in-person psychotherapy.

A key strength of this research is that we have identified key barriers and facilitators across three key and distinct participant groups of patients, providers, and stakeholders, which strengthens our findings by producing recommendations and insights from diverse perspectives [35]. Our provider and patient-centered approach is critical to the delivery of all healthcare interventions [36]. In addition, our systematic application of rigorous qualitative methods of barriers and facilitators produced practical and clearly explained suggestions that will help inform best practices for researchers and practitioners in outpatient treatment settings, including those that cannot offer their services remotely.

## 5. Conclusions

This paper drew on multistakeholder, qualitative data to examine the relevant factors to resuming in-person psychotherapy sessions with perinatal patients. This study found that COVID-19 has fostered a number of potential barriers to resuming in-person psychotherapy, and has likely exacerbated existing barriers, such as finding childcare and the burden of having to plan and negotiate competing priorities. However, the most reported facilitators focused on those that mediate COVID-19-specific barriers. Although facilitators such as implementing safety protocols are in-place in healthcare settings, our results suggest that there is a gap between the existence of such protocols and how perinatal patients might assess their level of risks when asked to access healthcare locations for in-person psychotherapy. Despite existing safety protocols and low rates of COVID-19 transmissions in hospitals, there is the perception that perinatal patients will nonetheless feel less safe and our findings suggest that this belief is likely to be attributed to perinatal patients who may feel particularly vulnerable while pregnant or fearful of exposing their newborn. Future interventions to facilitate resuming in-person sessions should focus on compassionate, patient-centered strategies and be aware of the need to address key barriers to resuming in-person therapy, in which providers and researchers take seriously the potential for ongoing risk aversion among perinatal patients.

For perinatal patients who are already experiencing depression and anxiety, reluctance to participate in in-person psychotherapy and clinical trials as the pandemic abates may be more acute. Our findings are benefited by the consensus between our three participant groups. Our findings highlight critical, feasible facilitators for resuming in-person psychotherapy: the need to implement safety protocols and develop clear, accessible, and patient-centered strategies to communicate to patients and research participants the many steps being taken to maintain their health and safety, as well as to find straightforward solutions, such as holding flexible and extended session times for this population.

## Figures and Tables

**Table 1:** Participant demographic characteristics.

Participant Demographics	Frequency (%) unless Otherwise Indicated
**Perinatal participants (*n* = 23)**	
**Age**	
Mean and range	32.0 (20–40)
**Location**	
Canada	14 (60.9)
United States	9 (39.1)
**Race/ethnicity**	
White	12 (52.2)
Other	9 (39.1)
Prefer not to answer	2 (8.7)
**Marital Status**	
Married or stable relationship	19 (82.6)
Single or dating	3 (13.0)
Prefer not to answer	1 (4.3)
**Employment**	
Maternity Leave	8 (34.8)
Full-time employment	6 (26.1)
Part-time employment	3 (13.0)
Unemployed	3 (13.0)
Other	3 (13.0)
**Highest Level of Education**	
High School or College/Trade School	5 (21.7)
University (undergraduate degree)	8 (34.8)
University (graduate degree)	10 (43.5)
**Household income**	
$0–$39,999	4 (17.4)
$40,000–$79,999	4 (17.4)
$80,000 or more	13 (56.5)
Prefer not to answer	2 (8.7)
**Number of children**	
No children, pregnant	12 (52.2)
1 child	8 (34.8)
2 children	3 (13.0)
**Providers (*n* = 28)**	
**Age**	
Mean and range	44 (41.3 to 46.6)
**Location**	
Canada	12 (42.9)
United States	16 (57.1)
**Provider Type**	
Specialists providers (SP)	13 (46.4)
Non-specialists providers (NSP)	15 (53.6)
**Gender**	
Female	26 (92.9)
Male	2 (7.1)
**Stakeholders (*n* = 18)**	
**Location**	
Canada	9 (50.0)
United States	9 (50.0)
**Stakeholder Type**	
Community-based (patient advocates, clinicians, community partners)	9 (50.0)
Hospital-based (psychiatrists, hospital administrators, and clinicians)	9 (50.0)

**Table 2:** Reported barriers to resuming in-person psychotherapy sessions, *n* (%).

Key Themes	Perinatal Participants *n* = 23	Provider Participants *n* = 28	Stakeholder Participants *n* = 18
**COVID-19-specific**			
Concerns about virus exposure in the hospital	17 (73.9)	20 (71.4)	14 (77.8)
Concerns about virus exposure on public transit	6 (26.1)	7 (25.0)	9 (50.0)
Needing to wear a mask during sessions	3 (13.0)	7 (25.0)	9 (50.0)
**General**			
Lack of childcare	10 (43.5)	10 (46.4)	12 (66.7)
Transportation (traffic, drive-time, lack of vehicle, cost of parking)	11 (47.8)	7 (25.0)	10 (50.0)
Planning and time constraints are onerous for busy new parent	8 (34.8)	13 (35.7)	5 (27.8)

**Table 3:** Reported facilitators to resuming in-person psychotherapy sessions, *n* (%).

Key Themes	Perinatal Participants *n* = 23	Provider Participants *n* = 28	Stakeholder Participants *n* = 18
**COVID-19-specific**			
Implementing and communicating robust safety protocols	11 (47.8)	11 (39.3)	13 (72.2)
Conducting sessions at offsite locations, not so deep within the hospital, or in larger rooms	4 (17.4)	9 (32.1)	8 (44.4)
**General**			
Providing incentives	8 (34.8)	6 (21.4)	2 (11.1)
Childcare and flexible scheduling for sessions	9 (39.1)	0 (0.0)	3 (16.7)

## Data Availability

The dataset (which includes individual transcripts) is not publicly available due to confidentiality policies and agreements.

## Supplementary Materials

The following are available online at https://www.mdpi.com/article/10.3390/ijerph182212234/s1, File S1: Perinatal participant interview questions, File S2: Demographic baseline questionnaires.
