# Supplementary material for: Barriers and Facilitators to Resuming In-Person Psychotherapy with Perinatal Patients amid the COVID-19 Pandemic: A Multistakeholder Perspective
Source: Int J Environ Res Public Health. 2021 Nov 22;18(22):12234. doi: 10.3390/ijerph182212234 (PMC8619135; doi:10.3390/ijerph182212234)
Supplement: Supplementary file 1 [file ijerph-18-12234-s001.zip › ijerph-1452012-supplementary.pdf]

International Journal of
Environmental Research and Public Health

MDPI

Appendix A:

Perinatal participant interview questions:

1. In your opinion, what are some of the barriers for future study participants to come to the hospital for in person therapy sessions?

2. What would make it easier for other women like yourself to feel comfortable coming to the hospital for in-person therapy sessions when we resume in-person sessions? (Probe: what would facilitate perinatal participants ability/ comfort to come to in-person sessions or changes could be introduced/what could be done differently?)

Provider interview questions:

1. In your opinion, what might be some potential barriers for future SUMMIT study participants (perinatal women) to come to the hospital for in person therapy sessions?

2. In future, if the SUMMIT study wanted to resume in-person sessions, what do think would make it easier for perinatal women to feel comfortable coming to the hospital for in-person therapy sessions?

Stakeholder interview questions:

1. In your opinion, what might be some potential barriers for future SUMMIT study participants to come to the hospital for in person therapy sessions?

2. In future, if the SUMMIT study wanted to resume in-person sessions, what do think would make it easier for perinatal women to feel comfortable coming to the hospital for in-person therapy sessions?

Appendix B: Demographic baseline questionnaires

Perinatal participant demographic questions

1. **What is your age?** ______________________________(years)

2. **As you know, people living in Canada and the United States come from many different countries and cultures. Is there a particular ethnic group to which you belong?**

   a) American Indian/Alaskan Native* (*if in the US)
   b) First Nations/Aboriginal
   c) Hispanic
   d) Asian
   e) Black/African American
   f) Hawaiian/Pacific Islander
   g) White
   h) Multi-race
   i) Other: ____________________
   j) Not wish to answer

3. **What is the highest level of education you completed?**
   a) Elementary School
   b) High School
   c) College/Trade School

d) University (undergraduate degree)
e) University (graduate degree)
f) Not wish to answer

4. What is your marital status?
a) Married or stable relationship
b) Dating/Uncommitted Relationship
c) Widowed
d) Single
e) Separated
f) Divorced
g) Other
h) Not wish to answer

5. What is your current employment/work status?
a) Full-time
b) Part-time
c) Retired
d) Disabled
e) Full-time student
f) Part-time student
g) Homemaker
h) Unemployed
i) Maternity Leave
j) Other: ________________(specify)

6. What is your annual household income before taxes?
a) $0 - $19,999
b) $20,000 - $39,999
c) $40,000 - $59,999
d) $60,000 - $79,999
e) $80,000 or more
f) Not wish to answer

7. How many children do you have?
a) 0
b) 1
c) 2
d) 3
e) 4
f) 5 or more

Provider demographic questions

1. **What is your gender?**
   a) Male
   b) Female
   c) Do not wish to answer

2. **Age:**
   ________ / Do not wish to answer

3. **What is your designation?**
   a) Mental Health Specialist
   b) Non-Mental Health Specialist
